# Stability of dynamic radiomics features in cardiac MRI under noise

**DOI:** 10.1093/ehjimp/qyag041

**Published:** 2026-03-13

**Authors:** Mike D Klaus, Fabian Laqua, Bettina Baeßler, Markus J Ankenbrand

**Affiliations:** Chair for Computational and Theoretical Biology, Julius-Maximilians-University Würzburg, Klara-Oppenheimer-Weg 32, 97074 Würzburg, Germany; Department of Diagnostic and Interventional Radiology, University Hospital Würzburg, Oberdürrbacher Straße 6, 97080 Würzburg, Germany; Department of Diagnostic and Interventional Radiology, University Hospital Würzburg, Oberdürrbacher Straße 6, 97080 Würzburg, Germany; Chair for Computational and Theoretical Biology, Julius-Maximilians-University Würzburg, Klara-Oppenheimer-Weg 32, 97074 Würzburg, Germany; Department of Bioinformatics, Julius-Maximilians-University Würzburg, Biocenter, Am Hubland, 97074 Würzburg, Germany

**Keywords:** radiomics, magnetic resonance imaging, heart, random noise, feature stability

## Abstract

**Aims:**

Radiomic studies on cardiac MRI mainly focus on images from distinct time points rather than considering the system’s dynamic nature. Recent studies have shown that radiomic features exhibit considerable variation across the cardiac cycle and that dynamic features can improve classification accuracy in downstream tasks. However, it is unclear whether the dynamic temporal evolution of radiomic features is sufficiently stable in the presence of noise. In this work, we evaluate the stability of radiomic feature curves of cine CMR images under noise.

**Methods and results:**

We extracted 910 radiomic features from all time points of cine CMR images of 115 subjects from three cohorts with various levels of artificially added noise. The stability of feature curves is evaluated based on pairwise normalized mean absolute errors, and features are ranked by their stability. Feature stability, measured by mean pairwise MAE, ranged from near 0 to over 20, with most features showing values below 2.5. Stability rankings showed moderate consistency across subjects (median Spearman correlation coefficient of 0.58). Features from the grey level size zone matrix (GLSZM) category demonstrated lower stability compared to first-order features. Some features exhibited high sensitivity to noise level but remained stable across different noise realizations at the same level.

**Conclusion:**

Some radiomic feature curves remain stable under noise while showing variability over the cardiac cycle. These features are promising candidates for improving models using dynamic rather than static feature values.

## Graphical Abstract

**Graphical Abstract:**
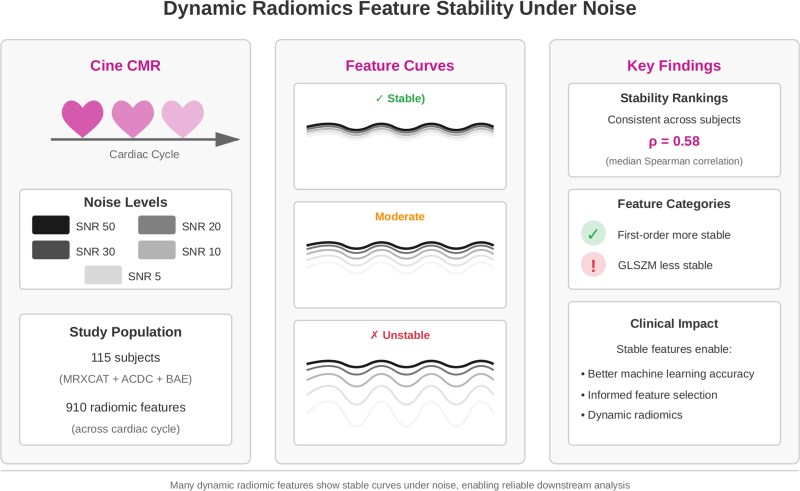

## Introduction

Radiomics is the process of quantifying textural information contained within medical images.^1^ The concept behind radiomics is that images generally contain information not visible to the human eye.^2,3^ Those features can be used to train machine-learning models for the detection, classification, and prognosis of various medical conditions.^4^ Radiomics has been successfully applied to different medical imaging modalities, including computed tomography,^5^ magnetic resonance imaging (MRI),^2^ and positron emission tomography.^6^ It is applied to a diverse set of medical tasks, including the prognosis in oesophageal cancer,^5^ finding cardiovascular risk factors,^7^ and differentiating between acute and chronic myocardial infarction.^8^

Most papers researching the application of radiomics in cardiac magnetic resonance (CMR) imaging only take the end-systolic (ES) or end-diastolic (ED) images of the cardiac cycle into account for feature extraction.^7–9^ However, there is evidence that radiomic features differ throughout the cardiac cycle.^10,11^ Features calculated on all time points of CMR images are more robust than features based on ES or ED images.^10^ Rather than combining features from all frames in a single value, it might be beneficial to consider values from all frames separately.^12^

The way a radiomic feature changes throughout the cardiac cycle could encode valuable information about certain cardiac conditions, especially in those where the dynamics and function of the heart play a vital role.^13^ We refer to radiomic studies that consider the temporal dynamics of a system as *dynamic radiomics* and the sequence of feature values as *feature curves*.

Generally, feature stability and repeatability are a primary concern.^14–16^ This limits the usage of radiomics as a prognostic and diagnostic tool since image acquisition settings and scanners differ in different hospitals.^17^ Hence, further research is necessary to explore possibilities to ensure reproducible features and thus allow clinical adoption for radiomics workflows.^18,19^ The stability problem might be exacerbated when adding a temporal dimension with additional inherent variation. To be useful for downstream tasks, radiomic feature curves must exhibit some stability under noise.^20^ So far, it is unclear whether radiomic feature curves of the left ventricular myocardium have this stability in cine CMR.

To answer this question, we analyse the impact of Gaussian noise on the stability of feature curves for more than 900 radiomic features on 115 subjects with four noise levels.

We hypothesize that some radiomic feature curves are insensitive to noise. Identifying those feature curves should help train more reliable machine-learning models to differentiate diseases in medical images.

Besides quantifying the stability of radiomic feature curves for this specific setting, we publish all code as an open-source Snakemake^21^ workflow, thus providing a framework for generally assessing feature curve stability for dynamic radiomics.

## Methods

### Study design

**Figure 1:**
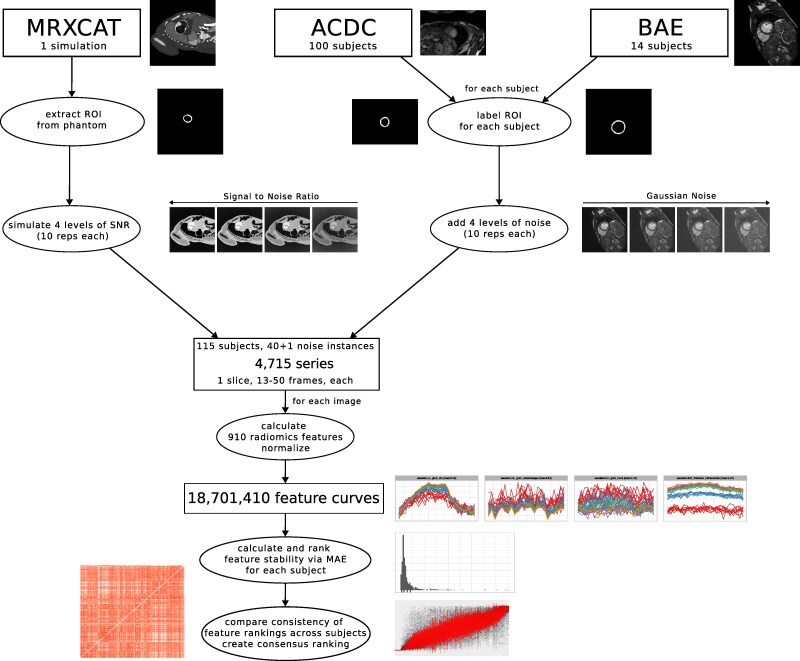
Overview of the data processing for the three data sources.

This study elucidates how stable the progression of radiomic features in the myocardium over the cardiac cycle (*feature curves*) is at different noise levels (*Figure 1*). A combination of simulated and retrospective data analysis is used. Data for this study comes from three sources: (i) simulations with MRXCAT, (ii) the public ACDC dataset, and (iii) data from a previous study by Baessler *et al.*^22^ (referred to as BAE). All cine time points of a single mid-ventricular short-axis slice are used for each subject. Radiomic features are calculated for every time point. The stability of the resulting feature curves under noise is examined.

### Study populations

The total study population (*n* = 115) consists of the MRXCAT phantom (*n* = 1), the patients from the ACDC dataset (*n* = 100), and the volunteers from the BAE dataset (*n* = 14). We refer to any individual in the study population as a subject, whether a phantom, patient, or volunteer.

#### MRXCAT

MRXCAT^23^ is a programme that creates realistic simulations of MRI images over the cardiac cycle via an XCAT^24^ phantom of the whole body. It simulates different aspects of MRI acquisition by considering physiological properties and MRI parameters. In contrast to actual MRI, we can precisely control the signal-to-noise ratio (SNR). We used MRXCAT to simulate images with the included breath-hold phantom and different SNRs. Five replicates for each SNR value (5, 10, 20, and 30) were generated. All other parameters were kept fixed at the default value for all simulations [i.e. repetition time (TR) 3.0 ms, echo time (TE) 1.5 ms, flip angle (FA) 90°]. Another simulation with SNR 50 is considered the reference. The phantom consists of a single slice of 1024 × 1024 pixels over 24 time points along the cardiac cycle. The field of view for the simulations was restricted to the central 512 × 512 pixels. The left ventricular myocardium, excluding papillary muscles, is defined as the region of interest (ROI) and precisely extracted from the phantom.

#### ACDC

The ACDC dataset was published as part of the Automatic Cardiac Detection Challenge. It consists of cine MRI scans of 150 subjects from clinical exams at the University Hospital of Dijon (France).^25^ We used data from the 100 subjects of the training set. These include 20 individuals with normal cardiac anatomy and function (NOR, defined by the authors of ACDC as ejection fraction greater than 50%, wall thickness in diastole lower than 12 mm, LV diastolic volume below 90 mL/m^2^ for men and 80 mL/m^2^ for women, RV volume less than 100 mL/m^2^ and RV ejection fraction above 40%, normal visual analysis of the segmental LV and RV myocardial contraction) and 20 cases each of myocardial infarction (MI), hypertrophic cardiomyopathy (HCM), dilated cardiomyopathy (DCM), and abnormal right ventricle (ARV). The left ventricular myocardium was segmented with nnU-Net^26^ for each subject, and a central slice was selected for further analysis. The number of frames varies between 12 and 35.

#### BAE

The full BAE dataset consists of 30 healthy volunteers (age 36 ± 13 years, heart rate 62 ± 13 min^−1^), of whom 15 subjects were initially selected for this study. One subject had to be excluded because automatic segmentation failed, resulting in 14 subjects (8 male, 6 female).^22^ The study received ethics approval from the ethics committee of the medical faculty of the University of Cologne (reference number 13-324). Written informed consent, including consent for publication, was given by all volunteers. All subjects were healthy and did not suffer from any abnormal cardiac conditions (inclusion criteria: (i) no significant medical history, (ii) no signs of inflammation, (iii) no symptoms indicating cardiovascular dysfunction, and (iv) normal cardiac dimensions and function confirmed by cine CMR). Volunteers with a history of inflammatory disease, including the common cold virus, in the last four weeks before the scans, were excluded. We used short-axis slice stacks with 50 phases per cardiac cycle for each subject. The examinations were conducted on a 1.5T scanner (Achieva 1.5T, Philips Medical Systems, Best, The Netherlands). The imaging parameters used for the 1.5 T short axes stacks were TR 28 ms, TE 1.4 ms, FA 60◦, field of view 343 × 380 mm^2^, matrix 256 × 256, slice thickness 8 mm, and 50 cardiac phases. Each subject’s left ventricular myocardium was segmented with a pre-trained artificial neural network,^27^ and a central slice was selected for further analysis.

### Noise

For the MRXCAT phantom, a reference (SNR = 50) and five replicates for each of the four noise classes with precisely defined SNR (30, 20, 10, and 5) were directly simulated. The reference can be considered almost noise-free, while the other SNR levels cover a range from mild to moderate to high noise.

For the real subject data (BAE and ACDC), images with different SNRs were derived by adding artificial (Gaussian) noise with TorchIO.^28^ The maximum SNR for each subject was given with the original images. Adding increasing amounts of artificial noise gradually reduces the SNR. Using TorchIO’s RandomNoise function with different values for standard deviation (std), 10 replicates of MRI images for four selected noise levels were generated. Values for std include 0.010, 0.020, 0.030, and 0.040. These were chosen by visual inspection of the generated noise levels and intended to be comparable to the noise of the different SNR classes generated in the MRXCAT simulations. So for each subject, there were 41 noise instances, consisting of the original image plus four noise classes with ten replicates, each.

### Radiomic feature curves

Texture, intensity, and shape analysis on all subjects was performed via AutoRadiomics,^29^ a radiomics tool for automatic pre-processing, feature selection, modelling, and model evaluation, which uses pyradiomics^30^ for feature extraction. For each subject, all frames of a single central slice with left ventricular myocardium as ROI were used. The default pre-processing of AutoRadiomics for MRI was applied to the data. Features were extracted on raw, wavelet-filtered, and Local Binary Pattern 2D (LBP2D) pre-processed data in six categories: first-order intensity statistics, grey level co-occurrence matrix, grey level run length matrix, grey level size zone matrix (GLSZM), grey level dependence matrix, neighbourhood grey tone difference matrix. We excluded shape features as they only depend on the ROI, which was the same for all noise levels. In total, 910 filter-feature combinations were extracted for every frame of each noise instance and subject. The corresponding feature values across frames are combined into feature curves, leading to feature curves for the 115 subjects with 41 noise instances and 910 features each (*Figure 1*).

### Feature curve stability

For comparability, all feature curves for each feature and subject (i.e. across all noise instances) were normalized such that the curve with the least noise (i.e. SNR 50 for MRXCAT and no noise added for ACDC and BAE) had a mean of 0 and a standard deviation of 1. A total of 45 features showed no variation over the cardiac cycle and were excluded during the normalization step, as features with zero standard deviation cannot be meaningfully normalized.

We calculated the pairwise distance of all noise instances for each feature and subject. The distance was measured as the mean absolute error (MAE) of normalized feature curves, calculated with the package similarity measures.^31^ The subject-level stability for each feature under noise is given by the mean of pairwise MAE values, with lower values indicating higher stability (0 would be the theoretical maximum stability). Ordering features by these noise values results in a subject-specific feature ranking. To assess the consistency of these rankings, we calculated pairwise correlations of all subjects using the Spearman correlation coefficient. A consensus ranking across subjects was achieved by ordering features by their median rank. A further measure of stability was calculated by restricting the pairwise distances to feature curves of the same noise level. This allows us to identify features sensitive to differences in the amount of noise but not the specific noise itself.

To understand the impact of feature stability on machine-learning model performance, we trained individual decision tree models for each feature with tidymodels (version 1.4.1)^32^ and the rpart engine (version 4.1.23).^33^ We used the ACDC data, subdividing the 100 patients into 60 for training, 20 for validation, and 20 for testing. Each of the five classes (NOR, MI, DCM, HCM, and ARV) was balanced across sets with 12 training, 4 validation, and 4 test cases per class. We used normalized feature curves uniformly subsampled to 12 frames, including all noise instances for the test and validation sets and only original images for the test set. Features with a validation accuracy of at least 33% were considered informative, and their test-set accuracy was evaluated.

### Data availability statement

The whole analysis is implemented as a Snakemake^21^ workflow. All code is available via GitHub (https://github.com/BioMeDS/cmr-dynamic-radiomics-stability) and Zenodo (https://doi.org/10.5281/zenodo.15239556). Large files, including simulation data, result tables, and figures are available as dvcstore via Zenodo (https://doi.org/10.5281/zenodo.15239918). ACDC data is available through their website.^25^ BAE data is not publicly available.

## Results

**Figure 2:**
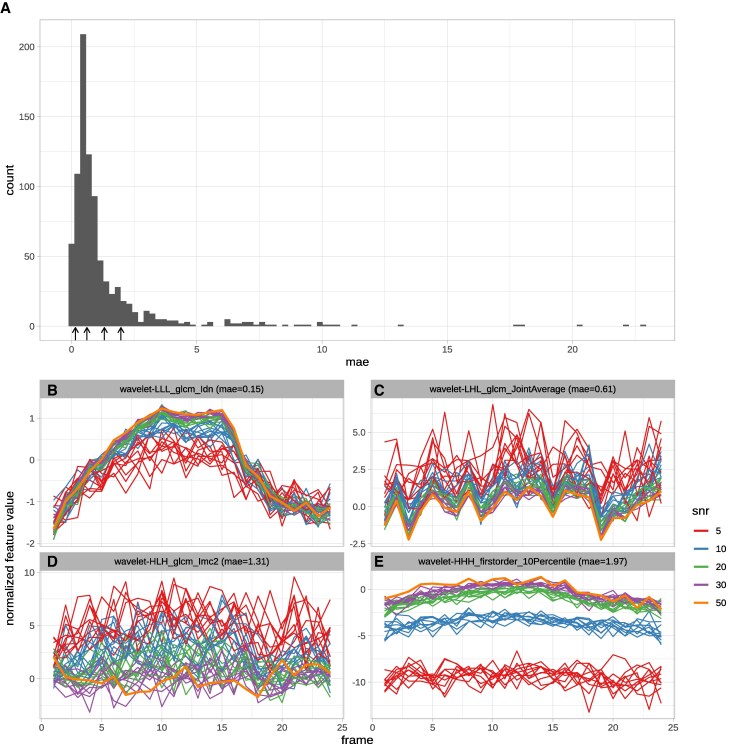
MAE score distribution for the MRXCAT simulation (*A*) and exemplary feature curves for a range of MAE values: wavelet-LLL_glcm_ldn with low mae (0.15, *B*), wavelet-LHL_glcm_JointAverage with intermediate mae (0.62, *C*), wavelet-HLH_glcm_lmc2 with high mae (1.28, *D*) and wavelet-HHH_firstorder_10Percentile with even higher mae (1.99) but with clearly visible separation of noise levels (*E*). Feature curves were processed and plotted via tidyverse^34^ and ggplot2^35^ in R.^36^

As expected, the radiomic features show variability over the cardiac cycle. The extent to which the resulting feature curves for the reference images (SNR 50, no additional noise) remain stable under noise varies across features and subjects. The stability of features for the MRXCAT phantom, as measured by the mean pairwise MAEs of the normalized curves (mpMAE), ranges from 0 to 24, with most features having values below 2.5 (*Figure 2A*). For mpMAE values below 1, the cases with lower SNR well reproduced the pattern of the reference feature curve. In contrast, the SNR 5 cases show the highest variability (*Figure 2B* and *C*). For values above 1, there are apparent deviations from the reference curve. Some curves show high variability around the original values (*Figure 2D*). In contrast, others have an explicit dependency on the amount of noise; thus, curves from the same SNR level are consistent, but they are shifted compared to other SNR levels, and the reference curve (*Figure 2E*). To quantify this, we calculated a noise-level dependent score as the mpMAE between all pairs of the same SNR/noise level.

**Figure 3:**
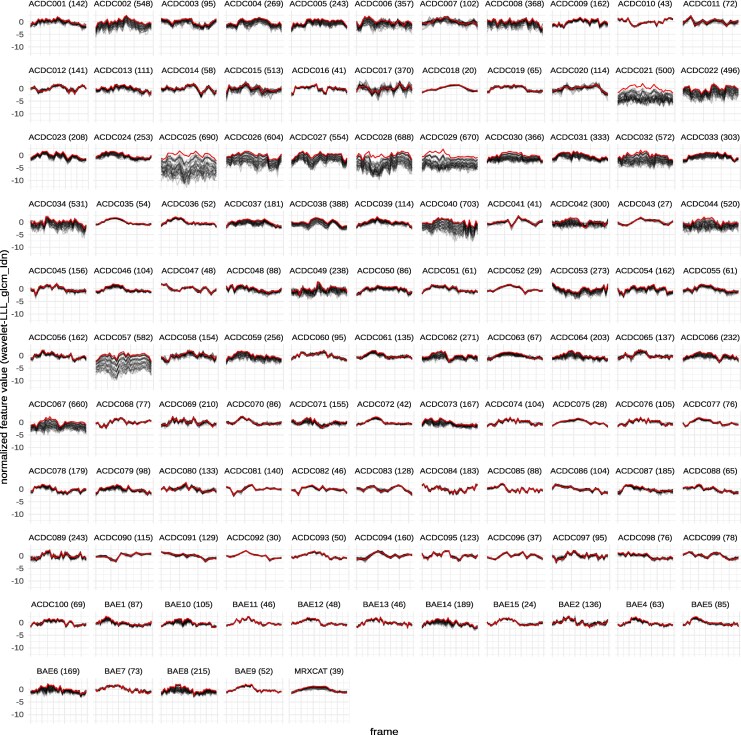
Feature curves for wavelet-LLL_glcm_ldn for all subjects. Reference curve (SNR50/no noise added) are shown in red, all noisy curves are shown in grey. The median rank for this feature is 366, the rank for each subject is printed in parentheses. Subjects are prefixed with the dataset they come from (ACDC, BAE, or MRXCAT). Plots for all features are deposited at https://doi.org/10.5281/zenodo.17752093.

Comparing all feature curves for a given feature across subjects shows that feature curves and their stability are similar for subjects from all three sets (*Figure 3* and Supplementary data online, *Supplementary File S1*).

**Figure 4:**
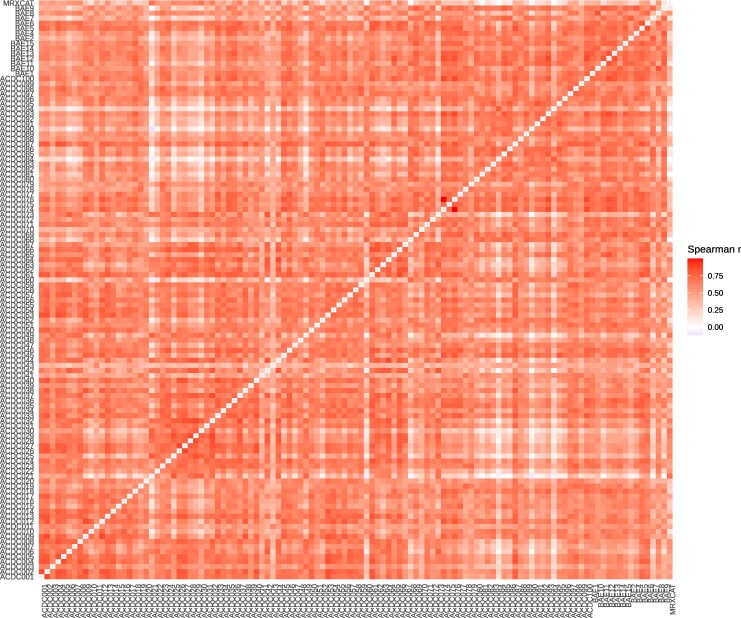
Pairwise Spearman correlation coefficient of feature stability scores (mae). White indicates no correlation, and dark red indicates a high correlation. The diagonal is empty as the correlation between a subject and itself is trivially 1.

**Figure 5:**
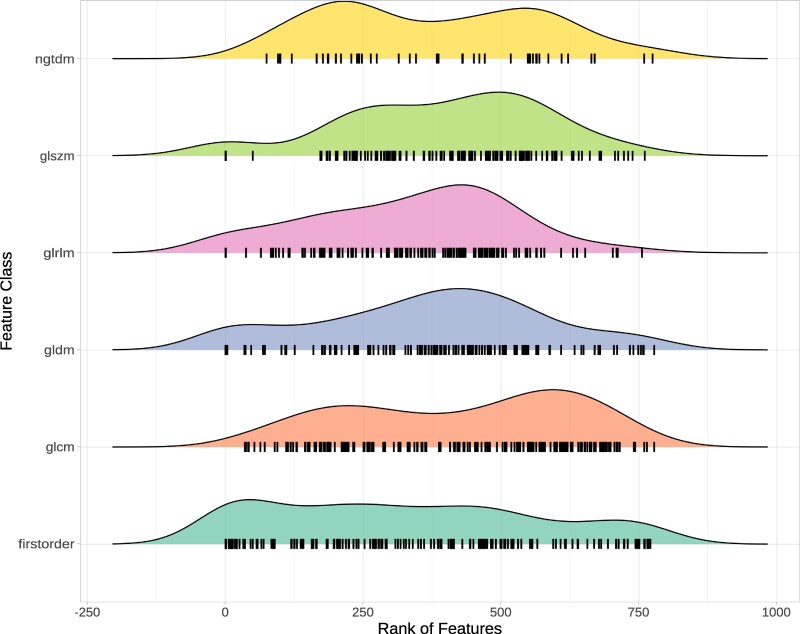
Distribution of consensus ranks across feature classes. Each feature is drawn as a vertical black bar with density plots drawn above.

The correlation coefficients range from −0.12 to 1.00, with a median Spearman correlation coefficient of 0.58 (*Figure 4*). Feature stability rankings were robust to differences in temporal resolution: sub-sampling the BAE dataset from 50 to 13 frames per cardiac cycle maintained Spearman correlations exceeding 0.89 between full and sub-sampled acquisitions (see Supplementary data online, *Figure S3*). A consensus ranking of all features was created by ordering features by their median rank (see Supplementary data online, *Figure S1*). In this global ranking, features from all categories show varying stability; however, features from category *glszm* tend to be less stable than features from category *firstorder* (*Figure 5*). Features derived from Local Binary Pattern 2D (LBP2D) pre-processing showed distinctive behaviour: approximately half exhibited no variation across the cardiac cycle and were excluded during normalization, while most remaining LBP2D features varied temporally but demonstrated complete insensitivity to noise (MAE = 0), maintaining identical feature curves across all noise levels and instances.

In addition to the consensus rank for each feature, the median mpMAE across subjects and the rank and median score for the noise-level dependent distance are reported (see Supplementary data online, *Table S1*). Comparing overall mpMAE and noise-level dependent mpMAE reveals features sensitive to the amount of noise but less so to the noise itself (see Supplementary data online, *Figure S2*).

More stable features tend to have higher classification accuracy on the ACDC data (see Supplementary data online, *Figure S5*). The Spearman correlation between rank and accuracy is −0.49 for informative features. The 15 most stable informative features reach a median test-set accuracy of 0.40, while the 15 least stable informative features have a median test-set accuracy of only 0.23.

## Discussion

As previously demonstrated, radiomic features vary across the cardiac cycle.^10^ Thus, considering the entire curve rather than limiting the analysis to distinct time points might increase the power. However, to be considered valuable inputs for predictive models, these feature curves must be robust to small perturbations, e.g. random noise.^20^ This work aimed to determine if the curves of any radiomic features are sufficiently stable under noise. We used data from 115 subjects and added four levels of noise. The resulting feature curves and mean pairwise MAE scores show that many features are robust to even high noise levels (*Figures 2A* and *B*, *3*).

In contrast, other features are susceptible to noise or at least the amount of noise (*Figure 2A, D*, and *E*). The observation that feature curves show different noise sensitivity led to the question of whether this noise sensitivity is subject-dependent. Indeed, mpMAE scores and ranks differ between subjects, in some cases considerably (e.g. *Figure 3*, ACDC025). However, overall, the rankings are consistent, with a median Spearman correlation coefficient of 0.59 across all pairwise comparisons (*Figure 4*). Correlations are similarly strong between subjects of the same and different cohorts (*Figure 4*). Therefore, our consensus scoring and ranking of radiomic features by their curve stability under noise (see Supplementary data online, *Table S1*) can serve as the foundation for feature selection in dynamic radiomics studies. An important consideration for feature selection in clinical applications is that stability and diagnostic value represent distinct, complementary dimensions. A feature may exhibit high stability yet lack clinical informativeness, or conversely, show lower stability but capture clinically relevant information across the cardiac cycle. Our study addresses the stability dimension but does not assess diagnostic or prognostic value, which requires validated disease cohorts with clinical endpoints. Importantly, we provide continuous stability scores rather than binary classifications, enabling researchers to balance these dimensions in their specific applications. For instance, a highly informative feature with moderate stability might warrant inclusion in a model, while a feature with lower informativeness would require correspondingly higher stability to justify its use. Our stability rankings (see Supplementary data online, *Table S1*) thus serve as one component of a comprehensive feature selection strategy that must ultimately weigh both stability and clinical performance. The stability of feature curves under noise is a prerequisite for further use in machine-learning models. However, it does not guarantee that these feature curves improve the performance compared to single feature values. This question can now be addressed in follow-up studies.

An interesting observation is that some features show high sensitivity to the amount of noise in the image while being relatively robust to different noise instances (*Figure 2E*, Supplementary data online, *Figure S2*). These features can be considered in studies with uniform noise levels, but should be avoided if some elements in the data set have higher levels of noise than others.

We demonstrated that models trained on feature curves of stable features reach higher classification accuracy on ACDC data than those trained on less stable features (see Supplementary data online, *Table S2*, Supplementary data online, *Figure S5*).

To allow users maximum flexibility in selecting the relevant features for their dynamic radiomics studies with LV myocardium on CMR, we provide overall and noise-level dependent rankings and scores for all radiomic features (see Supplementary data online, *Table S1*).

All our code and most of the data (except for BAE) are publicly available, thus allowing anyone to reproduce our findings and apply this approach to different datasets, regions of interest, or modalities. Hence, besides the concrete results for feature curve stability in LV myocardium in CMR, we provide a general framework for studying noise sensitivity in dynamic radiomics.

### Limitations

Several limitations of this study merit consideration. As with all observational studies, no causal conclusions could be drawn from the data. Hence, results from the non-experimental study design should be interpreted as hypothesis-generating. The scope of this study is limited to the stability of radiomic feature curves for a central slice of the left ventricular myocardium on CMR images to Gaussian noise. In particular, only within-subject stability was considered. This study is limited to simulated Gaussian noise, which may not fully capture the complexity of real MRI noise. In actual MRI acquisitions, noise follows a Rician distribution, particularly at low SNR levels, where the noise characteristics differ substantially from Gaussian noise. However, commonly used tools such as TorchIO support only Gaussian noise models. The use of Gaussian rather than Rician noise may affect the absolute values of our reported stability metrics. However, we expect that the relative ranking of feature stability would remain largely consistent across noise models, as all features are subjected to the same noise type. Nevertheless, validation with scan-rescan data, which would capture realistic MRI noise patterns along with other sources of variability such as patient repositioning and physiological variations, represents an important next step to confirm our findings under clinically realistic conditions.

Our analysis is restricted to a single mid-ventricular short-axis slice per subject. While we expect stability patterns to remain consistent across neighbouring mid-ventricular slices (confirmed by Spearman correlations >0.8 for adjacent slices in a test case), feature curves and their stability may differ in apical or basal regions. Extension to multi-slice or full 3D + t radiomics analysis represents an important direction for future research.

The results of this study establish the stability of feature curves under noise. We provide initial evidence linking stability to classification performance using single-feature decision tree models on the ACDC dataset, demonstrating that more stable features achieve higher accuracy. However, this validation is limited in scope. The observed median test-set accuracy of 40% for the most stable features reflects the use of individual features in isolation; higher accuracies would require multi-feature models, feature selection, optimized algorithms, and hyperparameter tuning, which are beyond the scope of this study. Comprehensive clinical validation comparing optimized models using stable dynamic features against conventional static features in disease cohorts represents an important next step. Furthermore, our study population consists exclusively of subjects with regular cardiac rhythms; the stability of feature curves in patients with arrhythmias, where dynamic radiomics may be particularly valuable, remains to be evaluated in future studies.

Our analysis assumes consistent segmentation quality across noise levels. In clinical practice, segmentation accuracy may deteriorate with increasing noise, potentially affecting feature stability through changes in the ROI rather than through intensity variations alone. To assess this effect, we evaluated segmentation stability using Dice scores across noise levels for one subject using misas^37^ (see Supplementary data online, *Figure S4*). While segmentation quality does decrease with noise, Dice scores remain above 0.7 even at the highest noise level examined (std = 0.04), suggesting a relatively modest impact within our studied noise range. Nevertheless, the interaction between segmentation variability and feature stability represents an important consideration that merits further investigation, particularly in clinical settings where automated segmentation methods may be less robust.

The variability of features and, thus, feature curves is expected to change in response to different acquisition methods and scanners and might require additional harmonization.^38^ While the reported feature curve stability might not be transferable to data from other hospitals, the described methodology can be applied to re-evaluate the features in these settings.

## Conclusion

We demonstrate that for many radiomic features, the way they vary along the cardiac cycle is stable under noise. This is a prerequisite for these feature curves to be considered as inputs to classification or regression models. We score and rank all features by overall and noise-level dependent stability based on one phantom and 114 subjects from two data sources. This table can be used to select features in follow-up studies utilizing dynamic radiomics. Exploring the potential incremental predictive value of feature curves over single features in supervised machine-learning tasks and medical conditions will be the subject of future research.

## Supplementary Material

qyag041_Supplementary_Data
