# Supplementary material for: Stability of dynamic radiomics features in cardiac MRI under noise
Source: Eur Heart J Imaging Methods Pract. 2026 Mar 13;4(1):qyag041. doi: 10.1093/ehjimp/qyag041 (PMC13007595; doi:10.1093/ehjimp/qyag041)
Supplement: qyag041_Supplementary_Data [file qyag041_supplementary_data.zip › Supplementary_Figure_legend.docx]

**Supplementary Figure 1.** Consensus ranking of features. Features are on the x-axis, ordered by median rank (median and mad shown in red), and individual ranks for each subject are shown as gray dots.

**Supplementary Figure 2.** Mean mae within noise level compared to mean mae across all noise levels. Each dot represents a feature. Range of x- and y-axis restricted to 0.0-1.0 (33 features are out of range and therefore not shown)

**Supplementary Figure 3.** Spearman correlation of feature stability of all BAE subjects with different temporal resolution: using full resolution of 50 frames and sub-sampled resolution of 13 frames per cardiac cycle.

**Supplementary Figure 4.** Sensitivity analysis of noise on segmentation accuracy for one BAE subject and time point using misas [35]. The Dice Score for the myocardium decreases with increasing noise levels. With the highest noise level used in this study (0.04), the Dice Score remains above 0.07. To avoid mixing the sensitivity to noise of segmentation models and of the radiomic features, the same mask was used for all noise instances.

**Supplementary Figure 5.** Test-set accuracy of decision trees built on individual informative (validation accuracy ≥ 33%) features. The blue line indicates the linear fit, with standard error bounds shown as a gray shaded area.
